# Incidence of and risk factors for Motor Neurone Disease in UK women: a prospective study

**DOI:** 10.1186/1471-2377-12-25

**Published:** 2012-05-06

**Authors:** Pat Doyle, Anna Brown, Valerie Beral, Gillian Reeves, Jane Green

**Affiliations:** 1 Faculty of Epidemiology and Population Health, London School of Hygiene and Tropical Medicine, London, WC1E 7HT, UK; 2 Cancer Epidemiology Unit, University of Oxford, Richard Doll Building, Roosevelt Drive, Oxford, OX3 7LF, UK

**Keywords:** Motor Neurone Disease, UK, Epidemiology, Incidence, Risk Factors, Smoking, Body Mass Index

## Abstract

**Background:**

Motor neuron disease (MND) is a severe neurodegenerative disease with largely unknown etiology. Most epidemiological studies are hampered by small sample sizes and/or the retrospective collection of information on behavioural and lifestyle factors.

**Methods:**

1.3 million women from the UK Million Women Study, aged 56 years on average at recruitment, were followed up for incident and/or fatal MND using NHS hospital admission and mortality data. Adjusted relative risks were calculated using Cox regression models.

**Findings:**

During follow-up for an average of 9·2 years, 752 women had a new diagnosis of MND. Age-specific rates increased with age, from 1·9 (95% CI 1·3 – 2·7) to 12·5 (95% CI 10·2 – 15·3) per 100,000 women aged 50–54 to 70–74, respectively, giving a cumulative risk of diagnosis with the disease of 1·74 per 1000 women between the ages of 50 and 75 years. There was no significant variation in risk of MND with region of residence, socio-economic status, education, height, alcohol use, parity, use of oral contraceptives or hormone replacement therapy. Ever-smokers had about a 20% greater risk than never smokers (RR 1·19 95% CI 1·02 to 1·38, p = 0·03). There was a statistically significant reduction in risk of MND with increasing body mass index (p_for trend_ = 0·009): obese women (body mass index, 30 kg/m^2^ or more) had a 20% lower risk than women of normal body mass index (20 to <25 Kg/m^2^)(RR 0·78 95% CI 0·65-0·94; p = 0·03). This effect persisted after exclusion of the first three years of follow-up.

**Interpretation:**

MND incidence in UK women rises rapidly with age, and an estimated 1 in 575 women are likely to be affected between the ages of 50 and 75 years. Smoking slightly increases the risk of MND, and adiposity in middle age is associated with a lower risk of the disease.

## Background

Motor neurone disease (MND) is a severe neurodegenerative disorder of the human motor system, characterised by the gradual death of upper and lower motor neurones and a resulting loss of motor function [1]. Incidence is extremely low below the age of 50, and rises thereafter, with lower rates in women than in men [2-4].

Little is known about the aetiology of MND. Around five to ten percent of cases are thought to be familial, and to date around 13 susceptibility genes and loci have been identified [5]. Few environmental risk factors have been identified, possibly reflecting the difficulties encountered in epidemiological studies of including sufficiently large numbers of cases and, in retrospective studies, knowing whether reported behaviours and exposures had changed as a result of early symptoms of the disease. The only factor for which there is some evidence of an association with MND risk is smoking [6-9]. This study makes use of a large cohort of 1.3 million UK women aged 56 years, on average, at the time of recruitment, with prospectively collected exposure information and with complete follow-up for almost 10 years to obtain incident cases of MND.

## Methods

### Study population

The Million Women Study (MWS) is a prospective cohort study of 1.3 million women who were recruited through National Health Service (NHS) breast screening centres in England and Scotland from 1996 to 2001 [10]. At recruitment, women completed a questionnaire (available at http://www.millionwomenstudy.org) asking about height, weight, alcohol consumption, and smoking, as well as socio-demographic details, reproductive history, and medical history. Height and weight were also directly measured in a randomly selected subsample of 2772 study participants [11]. Permission to conduct the study was granted by the Anglia and Oxford Multi-Centre Research Ethics Committee.

Study participants are followed up for cause-specific hospital admissions and deaths through linkage to centrally held computerised health records using their NHS number (a unique identifier) and other identifiers including date of birth and sex [10]. These linked records include the NHS central registries for deaths, cancers, and emigrations; the hospital episodes statistics (HES) in England [12] ; and the Scottish morbidity records (SMR) in Scotland [13]. The NHS central registries hold records of all registered deaths, including the cause of death and the date of death. The hospital admission databases contain a record of all NHS inpatient admissions from April 1997 in England and January 1981 in Scotland. The cause of death and diagnoses on admission to hospital were coded by using ICD-10 (the international classification of diseases, version 10). All study participants gave signed consent to be included.

### MND ascertainment and validation

According to the International Classification of Diseases (ICD-10) the code G12.2 is the generic code for MND, and includes diagnoses of amyotrophic lateral sclerosis, MND, progressive bulbar palsy, primary lateral sclerosis, and progressive muscular atrophy [14]. We classified women as having motor neuron disease (MND) if, during follow-up, they had (i) a hospital admission record with an ICD10 code of G12.2 and/or (ii) a death registration with any mention of ICD10 code G12.2.

During our investigations for this report we discovered an error in the WHO ICD-10 alphabetic coding index (Volume 3, page 429), where the code for Progressive Supranuclear Palsy was mistakenly given as code G12.2. Progressive Supranuclear Palsy is a degenerative disease of the basal ganglia and not a form of motor neurone disease (the correct ICD-10 code is G23.1, as given in Volume 1). We therefore reviewed the written text on all death certificates with a code G12.2. If the text on the death certicate mentioned Progressive Supranuclear Palsy, this was taken to indicate that the woman did not have MND, and that individual was excluded from the analysis. Overall, 8% of women who died with a code G12.2 on their death certificate were excluded as a result of this process.

For women with a G12.2 hospital admission record and who did not die in the follow-up period, we were unable to examine text (to exclude any remaining Progressive Supranuclear Palsy events) because written records were not available to us. A validation study was carried out to investigate such cases. Letters were sent to the General practitioners of a random sample of 89 MND cases ascertained from the HES dataset. General practitioners were asked to confirm the MND diagnosis, or provide information on alternative diagnoses, from medical notes. Sixty eight (76%) replied and 65 provided information about diseases in these women, among whom 91% (59) confirmed a diagnosis of MND. The remaining 9% (6) included 3% (2) with a diagnosis of Progressive Supranuclear Palsy, 5% (3) with another neurodegenerative disease, and one with colon cancer.

### Other data definitions

Address at recruitment was used to assign each woman to one of 4 regions in England and Scotland, roughly south to north: South West/Thames/Oxford/East Anglia; West Midlands/Trent; North West/North Yorkshire; Scotland. Socio-economic status was assessed using Townsend’s deprivation index, based on postcode of residence at the time of recruitment, and was categorized into quintiles [15]. Education was coded according the highest level of qualification achieved: college/university or equivalent; nursing/teaching or equivalent; A level; O level; none of these. BMI was calculated using height and weight reported at recruitment and participants were categorized as: underweight (<20 kg/m^2^); normal (20- < 25 kg/m^2^); overweight (25- < 30 kg/m^2^); obese class I (30- < 35 kg/m^2^); and obese class II & III (35+ kg/m^2^). Alcohol use reported at recruitment was recorded as units consumed on average per week: none; <7;7–14; 15 or more units per week. Smoking was also recorded at the time of recruitment and women were categorized as never smoker or ever smoker, the latter category being further split into current and past smoker. Parity was coded as nulliparous or parous, use of oral contraceptives as ever or never, and hormone replacement therapy (HRT) as never, past and current at time of recruitment.

### Analysis

Participants were excluded from analyses if they had a hospital admission record of MND before recruitment, or had a diagnosis of cancer (except non-melanoma skin cancer, ICD-10 C44) registered before recruitment. Woman-years were calculated from the date of recruitment to the date of hospital admission with MND, the date of death, emigration or the last date of follow-up, whichever came first. For analyses of MND incidence the last date of follow up was 31^st^ March 2008 for England and 31^st^ December 2008 for Scotland. For a small proportion (5%) of women recruited in England before 1 April 1997, we calculated person years from this date as hospital records were not available in England before this time.

Age-specific incidence rates were calculated as the number of new MND cases identified in the cohort divided by the person years of follow-up within 5 year age groups. Age-specific rates were compared to those reported in two recent publications from UK and Europe [3,4]. Crude cumulative risk for MND was estimated by summation of age-specific rates over the age range 50 to 74. This estimates the probability of MND up to age 75 in a hypothetical cohort who did not have MND at age 50.

Cox regression models were applied using the STATA computing package [16], taking attained age as the underlying time variable to obtain relative risk (RR) and corresponding 95% confidence intervals for socio-demographic, reproductive and behavioral factors, and BMI. Analyses were adjusted for year of birth (three groups), region, deprivation, smoking, alcohol use, HRT use, and BMI. When more than two categories were used in risk comparisons, results are presented as relative risk with corresponding floated confidence interval [17]. Analyses of trends in risk of MND by BMI at recruitment were carried out using mean self-reported BMI within the 5 BMI categories. This analysis was repeated using measured mean BMI (from the subsample [11]) within the five BMI categories.

To investigate the possibility of women having prodromal MND at the start of follow-up, all analyses were repeated using a lagging period of 36 months: follow up for each woman in the cohort was started 36 months after the date of recruitment.

## Results

A total of 1,319,360 women were followed up for an average of 9·24 years per woman. During 12 million woman-years of follow up, 752 women had a new diagnosis of MND: 695 had one or more hospital admission with MND, and 547 had MND recorded on their death certificate. The majority of women (65%) had MND recorded both at hospital admission and on their death certificate, and for only 8% (56) of women was death certification the only source of information about MND (Table 1). Ninety-one percent of a random sample of cases identified via hospital admission records had their diagnosis confirmed by their general practitioners.

**Table 1:** Source of information for incident MND cases in the study population

	**Incident MND cases**
Hospital admission data and death certificate	490 (65%)
Hospital admissions data only*	205 (27%)
Death Certificate only (any mention)**	57 (8%)
- underlying cause of death	54[95%]
- contributing cause of death	3 [5%]
**Total cases**	**752** (100%)

*To 31st March 2008 for England, 31st December 2008 for Scotland.

** To end December 2008.

**Figure 1:**
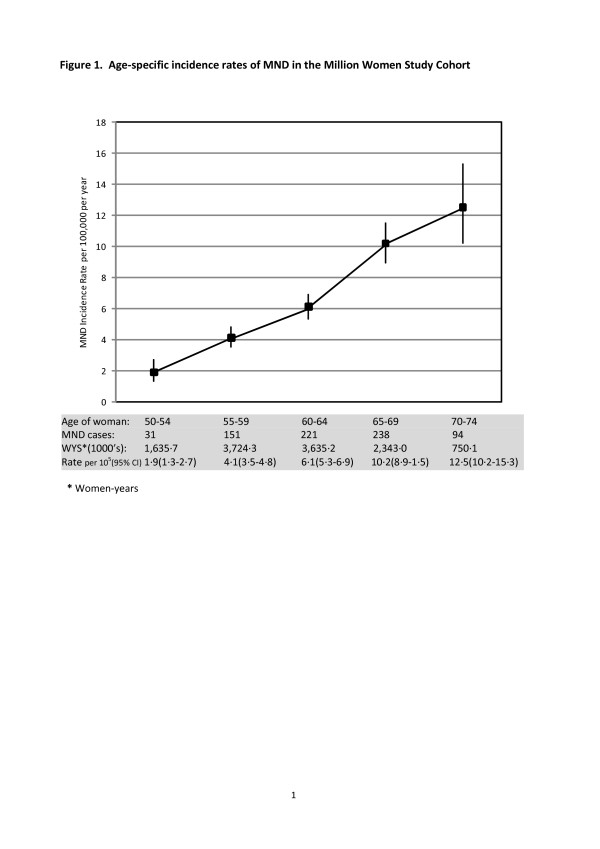
**Age-specific incidence rates of MND in the Million Women Study Cohort.** Age of woman: 50–54 55–59 60–64 65–69 70–74. MND cases: 31 151 221 238 94. WYS*(1000’s): 1,635·7 3,724·3 3,635·2 2,343·0 750·1. Rate per 10^5^(95% CI) 1·9(1·3-2·7) 4·1(3·5-4·8) 6·1(5·3-6·9) 10·2(8·9-1·5) 12·5(10·2-15·3). * Women-years.

Age-specific rates increased markedly with age, from 1·90 per 100,000 for women aged 50–54 to 12·5 per 100,000 for women aged 70–74 (Figure 1). The rate for women aged 75–79 was 17·4 per 100,000, based on only 17 cases. The estimated cumulative risk of MND between 50 and 74 years of age was 1·74 per 1000, equivalent to one in 575 women developing the condition over this 25 year period.

**Figure 2:**
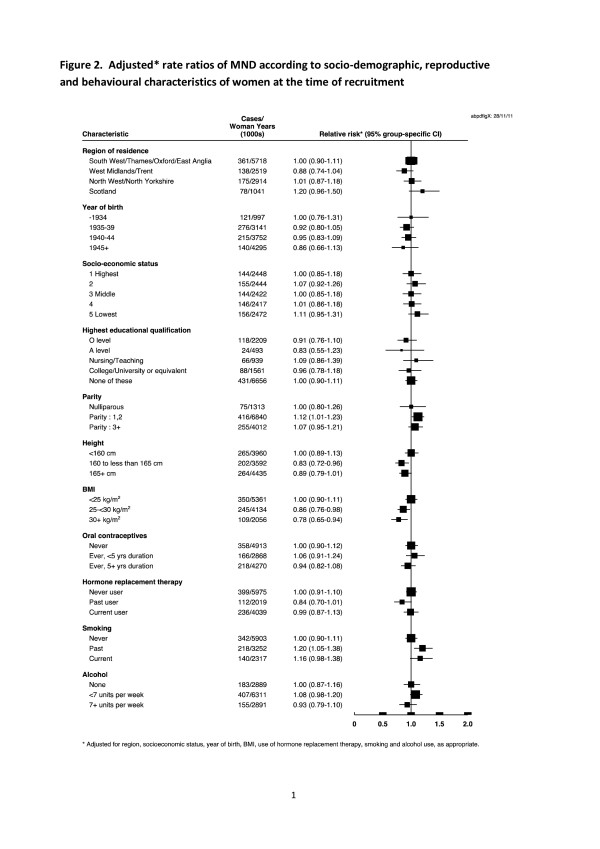
Adjusted* rate ratios of MND according to socio-demographic, reproductive and behavioural characteristics of women at the time of recruitment.

There was no significant trend in risk of MND with socio-economic status, education, parity, height, or alcohol use (Figure 2). Neither was there a significant association with use of oral contraceptives or hormone replacement therapy. Rates in Scotland were higher than the South of England but the confidence interval spanned one. There was no evidence of variation in risk by birth cohort. Having ever smoked was associated with a 20% increased risk (RR 1·19 95% CI 1·02-1·38, p = 0·026) compared to never smokers, and there was no difference in the increased risk between past and current smokers (Figure 2). Among current smokers the relative risks did not differ between those who smoked less than 15 cigarettes per day and those who smoked 15 or more cigarettes per day (RRs 1·20, 95% CI 0·94-1·55, versus 1·11, 95% CI 0·5-1·47).

There was a clear trend of a reduction in risk of MND with increasing BMI (Table 2, p_for trend_ = 0·009). Women classified as underweight at recruitment had 14% increased MND risk compared with those of normal BMI at recruitment, with a trend of decreasing risk as the obesity level increased. Women in the highest BMI category (BMI class II and III) at recruitment had a 30% reduction in risk compared with women with normal BMI at recruitment. Using means of self-reported BMI at recruitment, there was an estimated 2.3% reduction in risk of MND for each unit increase in BMI (RR per kg/m^2^ = 0·98, 95% CI 0·96 to 0·99, p for trend 0·01). Results were very similar when measured mean BMI from the validation sub-sample was used to estimate the mean BMI within each BMI category (RR per kg/m^2^ = 0·98, 95% CI 0·96 to 0·99, p for trend =0·011). The lagged analysis, excluding the first 3 years of follow-up, showed a similar trend of decreasing MND risk with increasing BMI, (RR per kg/m^2^ = 0·97, 95% CI 0·95 to 0·99, p for trend = 0·006). Around one third of woman-years in all the BMI groups were accrued in the first 3 years following recruitment. The proportion of MND cases incident in the first three years following recruitment were: 18% for cases underweight at recruitment; 17% for cases with normal BMI at recruitment; 25% for cases overweight at recruitment; 16% for cases who were obese class I at recruitment; and 32% for cases who were obese class II&III at recruitment.

**Table 2:** Relative risk of MND according to categories of BMI: Main analysis including all data and lagged analysis, excluding the first three years of follow-up

	**Main analysis**			**Excluding the first 3 years of follow-up**		
**BMI at recruitment Kg/m^2^**	**Number**	**Woman-year**	Adjusted Relative risk *	**Number**	**Woman-year**	Adjusted Relative risk *
	**cases**	**1000 s**	**(95% CI)**	**cases**	**1000 s**	**(95% CI)**
Underweight <20.0	32	431	1.14 (0.79-1.65)	26	286	1.14 (0.76-1.71)
Normal 20.0 to <25.0	318	4930	1.00 (0.89-1.12)	262	3296	1.00 (0.88-1.13)
Overweight 25.0 – < 30.0	245	4134	0.87 (0.74-1.03)	183	2757	0.80 (0.66-0.96)
Obese class I 30.0 - < 35.0	81	1462	0.82 (0.64-1.04)	68	972	0.84 (0.64-1.10)
Obese Class II & III 35.0 +	28	594	0.73 (0.49-1.07)	19	393	0.61 (0.38-0.97)

*Adjusted for region, deprivation, year of birth, use of hormone replacement therapy, smoking and alcohol use. ** 0.01 < p < 0.05.

## Discussion

The size and prospective nature of the Million Women Study are significant strengths of this study. MND has a relatively insidious onset so some behavior change may result from symptoms, making ascertainment of cases after the prospective collection of risk factor information critical. Case ascertainment using both hospital admission and death certification sources, combined with a very high level of diagnostic validation from general practioner records, adds to the study quality. And analyses excluding cases diagnosed in the first 3 years after recruitment provide further confidence in the robustness of the findings.

Prospective follow-up identified 752 MND cases incident during 9 years of follow-up (mostly in 1997–2008), making this the largest and most up-to-date study of incident MND cases in women yet reported in the UK. Incidence rates rose steadily with age giving a cumulative risk of 1 in 575 women developing the disease in the 25 years from age 50 to 75 years. Rates between 50 and 64 years were similar to recent population-based estimates reported from UK and Europe [3,4], and rates after age 65 were somewhat higher. There is some evidence of a continuing increase in MND mortality rates over time since 1950 [18-20], but no differences by birth cohort were observed here or in another UK study which spanned 15 years [4].

A recent review paper concluded that it was valid for epidemiological studies to use MND mortality data to estimate MND incidence within regions [21]. In the course of our investigations we discovered that 8% of the ICD-10 code G12.2 on death certificates erroneously included Progressive Supranuclear Palsy. However the proportion of death certificates coded to G12.2 with text indicating Progressive Supranuclear Palsy is not sufficiently large to invalidate analyses of trends in MND based on death certificates.

We did not find any association between MND and socio-economic status, education level, parity, height, or use of alcohol, oral contraceptives or HRT. This is the first time many of these factors have been investigated in MND research. The null findings are unlikely to be the result of low statistical power since this study was large and well powered to identify moderate associations should they be present.

We found a highly significant trend of decreasing risk with increasing BMI at recruitment, an observation which has not been reported before. Reverse causality, whereby those with undiagnosed disease at recruitment loose weight because of the condition, is a possible explanation for this observation. However some evidence here argues against this. The association remained the same in the lagged analysis which excluded all incident cases in the first three years after recruitment. The median time between onset of first symptoms and MND diagnosis is estimated to be around 12 months [22], so lagging analyses by 3 years should have allowed for the presence of undiagnosed disease at recruitment, at least to some extent. The cases diagnosed in the first three years following recruitment were not clustered disproportionately in the lower BMI groups, as would be expected if there was prodromal disease present at recruitment. Other possible methodological explanations include confounding and bias resulting from misclassification of BMI. Uncontrolled confounding is unlikely since so few risk factors for MND have been identified. With regards misclassification, BMI in the cohort was based on self reported height and weight, but comparisons with measured height and weight in this cohort, have shown that the effect of measurement error is small [23]. Also, we found similar trends from BMI trend analyses using average measured and reported category-specific BMI.

Ever smoking was associated with a modest 20% increased risk of MND compared to never-smokers. Among current smokers, relative risks did not differ by the amount smoked. A review of publications between 1990 and 2009 identified four good quality papers which were concordant for an effect of smoking on risk of ALS [6,7]. The author concluded that the level of evidence was sufficient for smoking to be considered an established risk factor for sporadic ALS [7]. A meta-analysis found a non-significant overall relative risk of 1·28 (95%CI 0·97-1·68) for smoking and MND risk [8], but after testing for a range of possible interactions, significant heterogeneity was found between studies, depending on the proportion of females in the study. The authors went on to conduct meta-regression taking the proportion of females into account, and the resulting model predicted a smoking-related risk of MND in females (RR 1·66, 97% CI 1·31-2·10) but not in males (RR 0·86, 95% CI 0·71-1·03). Their overall relative risk estimate in smokers, of 1·28, is similar to that found for women in our study, but their estimate of 1·66 for females is significantly greater than that found here; this could be because analyses looking for interactions in multiple subgroups can sometimes yield extreme results by chance [24].

## Conclusion

Overall, in this large prospective study we find a modest increased risk of MND in ever smokers and a significant decreasing risk for women with increasing adiposity.

## Competing interest

We declare that we have no conflicts of interest.

## Author contributions

All authors contributed to the design and execution of this work. PD, AB and VB prepared the report. All authors had an opportunity to contribute to the interpretation of the results and to the redrafting of the report, and all authors approved the final report.

## Pre-publication history

The pre-publication history for this paper can be accessed here:

http://www.biomedcentral.com/1471-2377/12/25/prepub
